# Bacterial Adhesion and Surface Roughness for Different Clinical Techniques for Acrylic Polymethyl Methacrylate

**DOI:** 10.1155/2016/8685796

**Published:** 2016-07-19

**Authors:** Lucas Costa de Medeiros Dantas, João Paulo da Silva-Neto, Talita Souza Dantas, Lucas Zago Naves, Flávio Domingues das Neves, Adérito Soares da Mota

**Affiliations:** ^1^Department of Fixed Prostheses, School of Dentistry, State University of Rio Grande do Norte, Caicó, RN, Brazil; ^2^Department of Dentistry, Federal University of Rio Grande do Norte, Avenida Senador Salgado Filho, s/n, Natal, RN, Brazil; ^3^LZNA Institute, Rua Alexandre Marquez 477, Uberlândia, MG, Brazil; ^4^Department of Fixed Prostheses, Occlusion and Dental Materials, School of Dentistry, Federal University of Uberlândia, Campus Umuarama, Avenida Pará No. 1720, Uberlândia, MG, Brazil

## Abstract

This study sought to assess the effect of different surface finishing and polishing protocols on the surface roughness and bacterial adhesion (*S. sanguinis*) to polymethyl methacrylates (PMMA). Fifty specimens were divided into 5 groups (*n* = 10) according to their fabrication method and surface finishing protocol: LP (3 : 1 ratio and laboratory polishing), NF (Nealon technique and finishing), NP (Nealon technique and manual polishing), MF (3 : 1 ratio and manual finishing), and MP (3 : 1 ratio and manual polishing). For each group, five specimens were submitted to bacterial adhesion tests and analyzed by scanning electron microscopy (SEM). Two additional specimens were subjected to surface topography analysis by SEM and the remaining three specimens were subjected to surface roughness measurements. Data were compared by one-way ANOVA. The mean bacterial counts were as follows: NF, 19.6 ± 3.05; MP, 5.36 ± 2.08; NP, 4.96 ± 1.93; MF, 7.36 ± 2.45; and LP, 1.56 ± 0.62 (CFU). The mean surface roughness values were as follows: NF, 3.23 ± 0.15; MP, 0.52 ± 0.05; NP, 0.60 ± 0.08; MF, 2.69 ± 0.12; and LP, 0.07 ± 0.02 (μm). A reduction in the surface roughness was observed to be directly related to a decrease in bacterial adhesion. It was verified that the laboratory processing of PMMA might decrease the surface roughness and consequently the adhesion of *S. sanguinis* to this material.

## 1. Introduction

Acrylic polymethyl methacrylates (PMMA) were one of the first materials used for dental provisional restorations. In order to form the polymer, PMMA prepolymerized fine particles are mixed with a liquid monomer resulting in polymers chains with properties and characteristics directly related to variables such as particle size, cross-linking agents, manipulation procedures, and monomer-polymer ratio [1]. PMMA provisional materials are more susceptible to bacterial retention and colonization than the materials used for final restorations due to the increased surface roughness and, generally, inferior fitting interfaces provided by the former [1, 2]. This is especially true when the restorations are worn for long periods. The adhesion of microorganisms to specific surfaces at the human oral cavity and the formation of dental plaque on these sites are the primary causes of several oral diseases that may consequently lead to unhealthy complications [1, 2].

The relationship between bacterial adhesion and the surface roughness of dental materials has previously been demonstrated [1–4]. It has been hypothesized that the subsequent proliferation of the initial adhering microorganisms is largely due to microbial mass increase during early plaque formation and may explain the importance of surface roughness in initial plaque formation [2]. Initial attachment of bacteria on roughened surfaces is aided by surface irregularities, where bacteria are protected from salivary flow and masticatory function, and can attach to more points at the substratum [5]. Further, the number of bacteria adherent to a specific surface is determined by some surface characteristics such as its hydrophobicity and surface charge [6]. Higher bacterial adherence on rougher surfaces occurs due to the presence of pits and grooves that reduce the influence of shear forces on the bacteria initially attaching to the surface [3, 7].

Polishing procedures generally increase the smoothness of the restoration surfaces. When polishing procedures are ignored, critical roughness may lead to further bacterial attachment. In addition, restorations poorly fitted may also increase bacterial adhesion to teeth and adjacent surfaces of the oral cavity [4, 6]. One method to reduce the poor fitting of the provisional restorations is a nonpressure technique, known as the brush or Nealon's technique, which was developed for intraoral border adjustment [8]. However, this technique may lead to an inadequate powder/liquid ratio, increasing the roughness of the restoration at critical interfaces between the teeth and the restorations.

Previous studies mostly examined streptococci bacteria, as they are “early colonizing bacteria” [9]. Due to the lack of information about* S. sanguinis* adhesion on PMMA restorations, this investigation aimed to assess the influence of finishing and polishing protocols on the surface roughness and bacterial adhesion to resin specimens made by different techniques. The null hypothesis was that the manufacturing techniques and the finishing and polishing protocols would not influence the surface roughness and bacterial adhesion to PMMA specimens.

## 2. Methodology

Fifty PMMA acrylic specimens (Duralay, Reliance Dental Mfg. Co., Worth, IL, USA) with 6.0 mm diameter and 2.0 mm height were made using a silicone rubber mold (Speedex Putty,* Coltene*-Whaledent, Altstätten, Switzerland) by 2 different techniques. Initially, all specimens were made following the manufacturer's directions 3 : 1 ratio in volume (powder : liquid). Then, twenty discs were worn down to 1.0 mm heights and rebased by Nealon's technique [9], using a brush (#00, Tigre S/A, São Paulo, Brazil) to apply the acrylic resin. Afterwards, the specimens were subjected to the finishing and polishing protocols as shown in Table 1. The specimens made by the manufacturer's directions (30) were subdivided into 3 groups (*n* = 10) as follows: finishing only (MF), finishing and manual polishing (MP), and finishing and laboratory polishing (LP). Specimens made by Nealon's technique (20) were subdivided into 2 groups of polishing, finishing only (NF), and finishing and manual polishing (NP). Finishing procedures were performed by means of a cylindrical flat end diamond rotary cutting instrument (HP 82 G, KG Sorensen, Barueri, Brazil). Manual polishing procedures were performed using 3 different instruments, a green silicon-carbide bur (GF 671.050HP, Edenta AG, Zurich, Switzerland), a light-gray polishing rubber (0371HP, Edenta AG), and a white rubber cup (KG Sorensen, Rio de Janeiro, Brazil). Each instrument was used for 30 seconds with petroleum jelly to minimize surface wear and to promote a smooth and shiny surface. Laboratory polishing procedures were performed at a bench vise using a soft brush with slurry of pumice and a felt disc with chalk powder for 30 seconds each.

For each group, five specimens were submitted to bacterial adhesion tests and analyzed by scanning electron microscopy (SEM) (LEO 435 VP; LEO Electron Microscopy Ltd., Cambridge, United Kingdom). Two additional specimens were subjected to surface topography analysis also by using SEM. The remaining three discs were subjected to surface roughness measurements, which were checked with a profilometer (Surftest SJ-301 Surface Analyzer; Mitutoyo Corp., Kawasaki, Kanagawa, Japan). Three parallel measurements were done on the surface of each specimen and their average was used to determine the mean surface roughness (Ra) value.

*S. sanguinis* was cultured in Brain Heart Infusion (BHI) for 24 h at 37°C on Petri plates. After a 24 h growth period, the infusion was adjusted to obtain bacterial concentrations of 10^9^ colony forming units per milliliter (CFU/mL) in sterile phosphate-buffered saline solution (PBS, 0.08 M, pH 7.8, Sigma-Aldrich, St. Louis, USA). The bacterial concentration was measured using the McFarland standard, which evaluates the optical density of the solution.

Five specimens from each group were randomly selected and incubated in bacterial suspension for 1 h at 37°C with 240 rpm of continuous shaking (Shaker-Adamo, Piracicaba, Brazil). The discs were then fixed with modified Karnovsky's solution (2% glutaraldehyde in filter-sterilized PBS buffer (0.1 M, pH 7.4)) at room temperature for 2 h and then rinsed 3 times for 15 min in PBS buffer (0.15 M). Next, a postfixation step was performed for 1 h with 1% osmium tetroxide in PBS buffer (0.1 M). This was followed by a final rinse with distilled water. Fixed discs were then dehydrated with progressive ethanol rinses. Samples were soaked in ethanol-water mixtures with increasing ethanol concentrations (30%, 50%, 70%, 80%, and 95%, successively) for 7 min each and then in pure ethanol for 5, 10, and 15 min, respectively. Dried samples were secured to metal holders with double-sided adhesive tape and then gold-sputtered (Desk II Sputtering, Denton Vacuum, Cherry Hill, USA). After that, specimens were analyzed by SEM. Images were usually acquired at 8 kV and 4Kx magnification. “Observation fields” were chosen randomly from the experimental surface of each PMMA disc to perform the CFU counting. The counts were averaged and the number of adherent CFUs per observation field was calculated.

The topographic analysis by SEM used similar parameters for the bacterial analysis except with 150x magnification. These samples were submitted to SEM preparation methods only.

All data were analyzed using a statistical package (SPSS 17.0, SPSS Inc., Chicago, USA). Kolmogorov-Smirnov test was used to check normality and Levene's test was used to calculate variance homogeneity (95% of confidence interval). After this one-way ANOVA was applied to detect the significance of differences between the groups (*α* ≤ .05). Tukey's test was used to compare groups. Group data were also verified by Pearson's correlation test (*r* ^2^).

## 3. Results

Significant differences were verified for the surface roughness among the groups (Table 2). The mean Ra values indicated that surface treatment and manufacturing technique influenced significantly the surface roughness (Table 3). LP specimens presented significantly smoother surfaces than other groups, while NF specimens presented rougher surfaces. NP and MP groups showed similar surface roughness and were smoother than MF.

**Figure 1:**
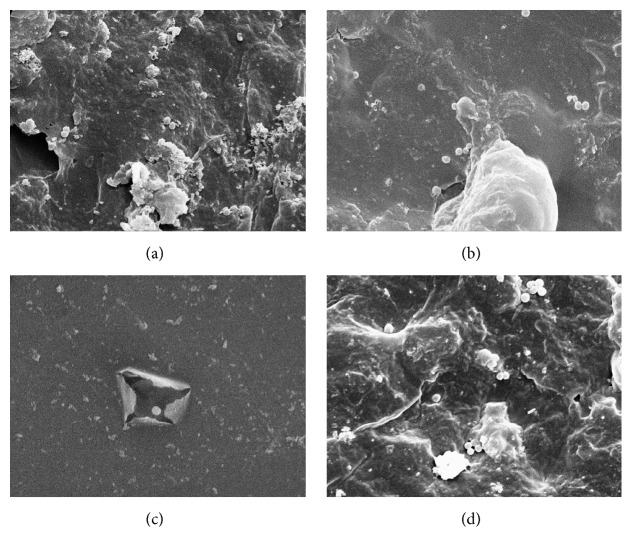
Sample SEM image of the “observation fields” at 4000x magnification.

*S. sanguinis* were found at all investigated surfaces, indicating bacterial adhesion rather than coaggregation, since isolated CFUs were detectable. One-way ANOVA also showed significant differences among the groups for bacterial adhesion (Table 4). The mean CFU values for the groups are presented in Table 5. Higher bacterial adhesion was observed for NF group (Figure 1). Lower bacterial adhesion was verified for LP group, which was similar to NP and MP groups, and significantly different to NF and MF groups. Polished groups, MP and NP, were similar to LP and MF groups but showed lower bacterial adhesion than NF group. A high positive correlation degree was verified between the surface roughness and the bacterial adhesion (*r* ^2^ = 0.808).

**Figure 2:**
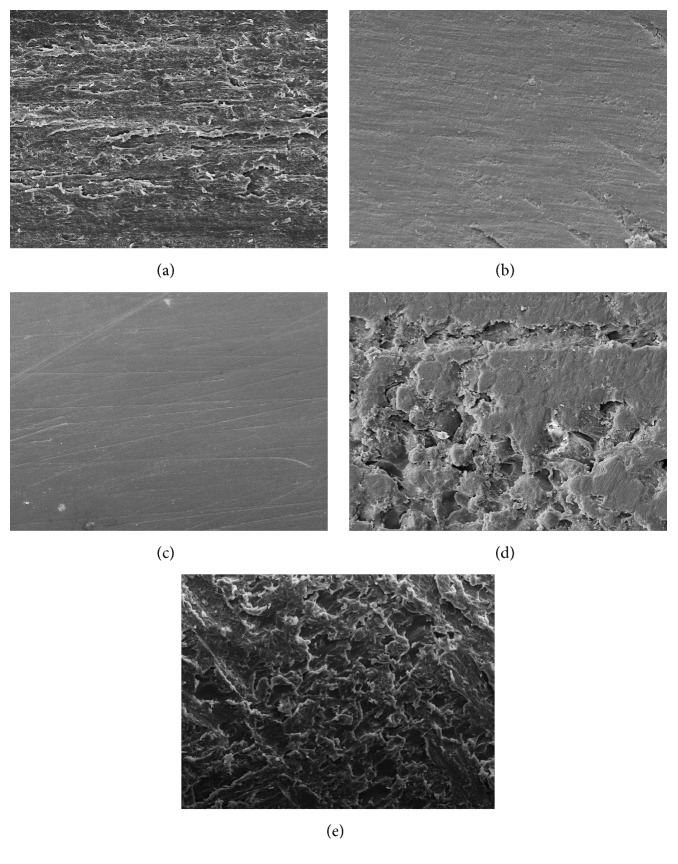
SEM images of sample topographies at 150x magnification. (a) MF; (b) MP; (c) LP; (d) NP; (e) NF.

The analysis of the surface topography showed that NF group presented more irregular surfaces, with marked grooves and pits, than the other groups. LP group showed smoother surfaces with less structural defects than the other groups. The polished groups, MP and NP, were similar to each other (Figures 2(a)–2(e)).

## 4. Discussion

The null hypothesis must be fully rejected. The results of the present study showed that different manufacturing techniques resulted in significant differences for surface roughness and bacterial adhesion. In addition, different finishing procedures produced significant differences regarding surface roughness and bacterial adhesion.

These findings are in accordance with previous studies that investigated the plaque formation on both provisional and nonprovisional restorative materials [1, 2, 5, 10–20]. These studies showed that increased surface roughness leads to an increased bacterial adhesion or plaque formation following its growth. The influence of the finishing protocols was verified by few studies [21, 22], and their results corroborate with the findings of the present investigation. Studies comparing different materials according to their surface roughness and bacterial adhesion also reported results that are in accordance with our findings [1, 2, 5, 10–22]. The present study found that surface roughness and bacterial adherence were influenced by manufacturing techniques and finishing/polishing protocols.

For both manufacturing techniques, bacterial adhesion was increased with the increased surface roughness, fact that was confirmed by Person's correlation. The presence of grooves and pits was more evident in the specimens made by means of Nealon's technique. These grooves and pits contributed to the high Ra values observed for these specimens (Table 3). Faltermeier et al. [18] found that the presence of internal defects (pits and grooves) provoked by the manufacturing process of composite specimens increased the Ra values. This could explain the higher Ra values verified for the specimens produced by Nealon's technique. However, when the specimens made by Nealon's technique were manually polished, there was no significant difference in surface roughness compared to the other manufacturing techniques.

There is a lack of information in the present literature comparing different finishing and polishing protocols for the same material. Usually, studies compare polished materials to verify the influence of the material in the surface roughness and bacterial adhesion [1, 2, 5, 9–21]. The present study compared manual and laboratory polishing protocols to specimens only finished using the same material. Therefore, this study demonstrated that laboratory finishing and polishing protocols resulted in a smoother surface than the other methods evaluated, leading to less streptococcal adhesion. Specimens finished but not polished and those made by Nealon's technique presented the roughest surfaces and the highest CFU values, indicating potentially critical issues for chairside application of these techniques in long term.

Although it cannot be generalized, since different types of bacteria exhibit particular adhesion properties to that of* S. sanguinis*, this stain is an early colonizer of the dental plaque, making our model suitable to test bacterial adhesion to biomaterials [20]. Mei et al. [23] reported that* S. mutans* adhered less strongly and its adhesion forces were less influenced by the surface roughness of the material than those of* S. sanguinis*. These findings are important to justify the use of* S. sanguinis*. For bacterial adhesion test the McFarland scale was used to standardize the quantity of CFU in the solution, and this allowed the comparison of the CFU amounts in the different groups.

Streptococci adhered stronger to rougher surfaces and, are therefore, more difficult to remove [23]. This is in agreement with the present results, which showed that more bacteria are adherent to rougher surfaces. Despite the comparison methods found in the literature, the correlation between surface roughness and bacterial adhesion related in this study was also found by other investigations [4, 21, 24]. This study indicates that surface roughness is the major factor in determining bacterial adhesion, which is consistent with other findings [4, 22–24]. Further studies evaluating the bacterial adhesion on different restorative materials in the presence of saliva under simulated clinical conditions such as brushing, mastication, and chemical cleaners will be of benefit.

## 5. Conclusions

Within the limitations of the present* in vitro* investigation, the following conclusions could be drawn:

1. Increased surface roughness was directly related to increased bacterial adhesion.
2. Specimens manufactured using Nealon's technique presented increased surface roughness and consequently increased bacterial adhesion.
3. Specimens finished but not polished showed increased bacterial adhesion compared to those finished and polished.
4. Specimens finished and polished by a laboratory protocol presented smoother surfaces and less bacterial adhesion.
5. Manual polishing of the specimens reduced the surface roughness and the bacterial adhesion.

## Figures and Tables

**Table 1:** Description of disc manufacturing technique, finishing.

Groups	Manufacturing technique	Finishing procedure	Polishing procedure
L (control)	3 : 1 ratio	Yes	Laboratory
MP	3 : 1 ratio	Yes	Manual
MF	3 : 1 ratio	Yes	None
NP	Nealon	Yes	Manual
NF	Nealon	Yes	None

**Table 2:** One-way ANOVA analysis of surface roughness.

Variation source	Sum of squares	df	Mean square	*F*	Sig.
Between groups	24.569	4	6.142	685.456	0.000
Within groups	0.090	10	0.009	—	—
Total	24.658	14	—	—	—

**Table 3:** Mean surface roughness values.

Group	Surface roughness (SD)^*∗*^
L (control)	0.09 (0.01) a
MP	0.52 (0.05) b
NP	0.61 (0.08) b
MF	2.69 (0.12) c
NF	3.23 (0.15) d

^*∗*^Alphabetic characters indicate groups for which the values are not significantly different.

**Table 4:** One-way ANOVA analysis of colony forming units.

Variation source	Sum of squares	df	Mean square	*F*	Sig.
Between groups	961.926	4	240.482	50.623	0.000
Within groups	95.008	20	4.750	—	—
Total	1056.934	24	—	—	—

**Table 5:** Mean values to colony forming units.

Group	Colony forming units^*∗*^
L (control)	1.56 (0.62) a
MP	5.36 (2.08) ab
NP	4.96 (1.92) ab
MF	7.36 (2.45) b
NF	19.60 (3.05) c

^*∗*^Same alphabetic character indicates groups for which the values are not significantly different.
